# Crystal Structure of a Conserved Hypothetical Protein MJ0927 from *Methanocaldococcus jannaschii* Reveals a Novel Quaternary Assembly in the Nif3 Family

**DOI:** 10.1155/2014/171263

**Published:** 2014-08-28

**Authors:** Sheng-Chia Chen, Chi-Hung Huang, Chia Shin Yang, Shu-Min Kuan, Ching-Ting Lin, Shan-Ho Chou, Yeh Chen

**Affiliations:** ^1^Department of Biotechnology, Hungkuang University, Taichung 433, Taiwan; ^2^Taiwan Advance Biopharm (TABP), Inc., Xizhi City, New Taipei City 221, Taiwan; ^3^School of Chinese Medicine, China Medical University, Taichung 40402, Taiwan; ^4^Institute of Biochemistry and Agricultural Biotechnology Center, National Chung Hsing University, Taichung 40227, Taiwan

## Abstract

A Nif3 family protein of *Methanocaldococcus jannaschii*, MJ0927, is highly conserved from bacteria to humans. Although several structures of bacterial Nif3 proteins are known, no structure representing archaeal Nif3 has yet been reported. The crystal structure of *Methanocaldococcus jannaschii* MJ0927 was determined at 2.47 Å resolution to understand the structural differences between the bacterial and archaeal Nif3 proteins. Intriguingly, MJ0927 is found to adopt an unusual assembly comprising a trimer of dimers that forms a cage-like architecture. Electrophoretic mobility-shift assays indicate that MJ0927 binds to both single-stranded and double-stranded DNA. Structural analysis of MJ0927 reveals a positively charged region that can potentially explain its DNA-binding capability. Taken together, these data suggest that MJ0927 adopts a novel quartenary architecture that could play various DNA-binding roles in *Methanocaldococcus jannaschii*.

## 1. Introduction

Given that numerous genomes of diverse organisms have been sequenced to date, it is not surprising that the number of hypothetical proteins with unknown functions has also steadily increased. Structures of hypothetical proteins can provide hints for deciphering their functions [1]. Since Nif3-like protein is widely distributed from bacteria to higher eukaryotes, this conserved hypothetical protein has been identified as a target from which the protein function can be inferred based upon a structural perspective [2].

The name of the Nif3-like superfamily was originated from the yeast Nif3, which was identified as an NGG1p-interacting protein in a yeast two-hybrid screen [3]. Sequence analysis of all reported Nif3-like proteins indicated that only the N- and C-terminal regions of the Nif3 proteins are highly conserved during evolution [4]. Studies of several eukaryotic Nif3 homologs have indicated that NIF3 family proteins are involved in transcriptional regulation [5]. For example, Akiyama et al. have demonstrated that murine Nif3 can interact with Trip15/CSN2 to function as transcriptional repressors [5]. Yet, the biological functions of Nif3 proteins in both prokaryotes and archaea kingdoms remain obscure.

To date, the tertiary structures of several bacterial Nif3 proteins have been determined by X-ray crystallography including those of SP1609 from* Streptococcus pneumonia *(2FYW), YqfO from* Bacillus cereus* (2GX8) [6], YbgI from* Escherichia coli* (1NMP) [7], SA1388 from* Staphylococcus aureus* (2NYD) [8], and TTHA1606 from* Thermus thermophiles* HB8(2YYB) [9]. These structures have all been observed to adopt a similar *α*/*β* structure, forming a toroidal hexamer. In most Nif3 structures, two metal ions were also found to stably exist within the cavity of the hexameric toroid, although the functional roles of these metal ions are still unclear.

To understand the structural differences between archaeal and bacterial Nif3 proteins, MJ0927 was selected as a target for structure determination. The crystal structure of MJ0927 determined at 2.47 Å revealed that MJ0927 folds into two interlinked *α*/*β* domains. Six protomers of MJ0927 are found to form a novel hollow cage-like hexamer, in contrast to the toroid-shaped hexamers formed by other members of the Nif3 family. Additionally, we found that MJ0927 is a DNA-binding protein exhibiting the ability to bind to both single-stranded DNA (ssDNA) and double-stranded DNA (dsDNA). These data indicate that MJ0927 is a new member of the Nif3 family.

## 2. Materials and Methods

### 2.1. Cloning, Expression, and Purification

The gene cloning, protein expression, purification, crystallization, and diffraction for the native MJ0927 have been previously reported [10]. For expression of selenomethionyl (Se-Met) labeled MJ0927, the* E. coli *BL21(DE3) host cells were cultured in M9 medium supplemented with 40 *μ*g mL^−1^ Se-Met at 37°C and induced by adding isopropyl *β*-D-thiogalactopyranoside to a final concentration of 0.5 mM when the cell density reached an OD_600_ of 0.6. Purification of selenomethionyl MJ0927 were performed using similar protocols as established for the native protein.

### 2.2. Crystallization, Data Collection, and Structure Determination

Purified selenomethionyl MJ0927 was concentrated to approximately 30 mg mL^−1^ for crystallization. Crystals were grown at 4°C using the sitting-drop vapor diffusion method by mixing 1 *μ*L of protein solution with 1 *μ*L of reservoir solution containing 5% PEG3350, 0.1 M sodium acetate, pH 5.0, 0.3 M sodium formate, 0.1 M ammonium sulfate, and 3% poly-*γ*-glutamic acid polymer (PGA-LM). Crystals were flash-cooled in the mother liquor supplemented with 25% glycerol as a cryoprotectant. X-ray diffraction data were collected using the BL13B1 beamline at the National Synchrotron Radiation Research Center (NSRRC) in Taiwan. A three-wavelength MAD data set was collected to solve the protein phases. Intensity data were scaled and reduced using the HKL-2000 program [11]. The Se-Met-labeled MJ0927 crystals were found to adopt space group P2_1_. The MAD dataset was used for solving the structure and an* ab initio* model was built by using the Autosol and Autobuild Wizards in the Phenix package [12], respectively. Twelve selenium sites were identified and the initial phases calculated from these sites were further improved by density modification. The resulting electron density map was readily interpretable and was used to build most of the MJ0927 structure, which was then refined by iterative manual model building in the Coot [13] and Phenix refinement module [14]. Finally, 244 residues out of a total of 249 amino acids could be clearly identified in the model of Se-Met MJ0927.

Crystals of native MJ0927 in the C222_1_ space group were obtained using the reservoir solution containing 0.1 M (NH_4_)_2_SO_4_, 0.3 M sodium formate, 0.1 M sodium acetate, 3% PGA-LM, and 20% MPD. The X-ray diffraction data of native MJ0927 were collected using the BL13C1 beamline at the NSRRC, Taiwan. The crystal structure was solved by the molecular replacement program Phaser [15] using the MJ0927 coordinates in P2_1_ form as the template. Three molecules of MJ0927 are present in each asymmetric unit. Subsequent refinement of the coordinates and individual B factors were carried out in the Phenix refinement module. Noncrystallographic symmetry restraints were included only in the initial stages of refinement. The *R* values were refined to 18.1% and 23.6% for the *R*_work_ and *R*_free_, respectively. The detailed crystallographic statistics were summarized in Table 1 [10]. Both coordinate sets have been deposited in the Protein Data Bank under the entries of 4IWG (C222_1_ form) and 4IWM (P2_1_ form).

### 2.3. Electrophoretic Mobility-Shift Assay (EMSA)

Two types of DNA were used for the DNA-binding experiments: a single-stranded 37-bp oligonucleotide and a double-stranded 37-mer oligonucleotide (5′-ATGTGAATCAGTATGGTTACTATCTGCTGAAGGAAAT-3′  and 5′-ATTTCCTTCAGCAGATAGTAACCATACTGATTCACAT-3′). These DNA were purchased from MDBio Inc. (Taiwan) and labeled by reaction with T4 polynucleotide kinase in the presence of [*γ*-^32^P]ATP. The purified His-tag-free MJ0927 was incubated with 25 nM ssDNA or dsDNA for 60 min, in a 10 *μ*L solution containing 50 mM Tris-HCl, pH 8.0, 100 mM NaCl, 5% glycerol, 100 *μ*M bovine serum albumin, and 2 mM Tris(2-carboxyethyl)phosphine at 37°C. The samples were then loaded onto a native gel of 5% nondenaturing polyacrylamide in 0.5× TB buffer (45 mM Tris-HCl, pH 8.0, and 45 mM boric acid). The DNA and protein-DNA complexes were separated by electrophoresis. Gels were exposed to phosphor storage screens and analyzed on a phosphorimager (Typhoon 9200, GE Healthcare).

## 3. Results and Discussion

**Figure 1:**
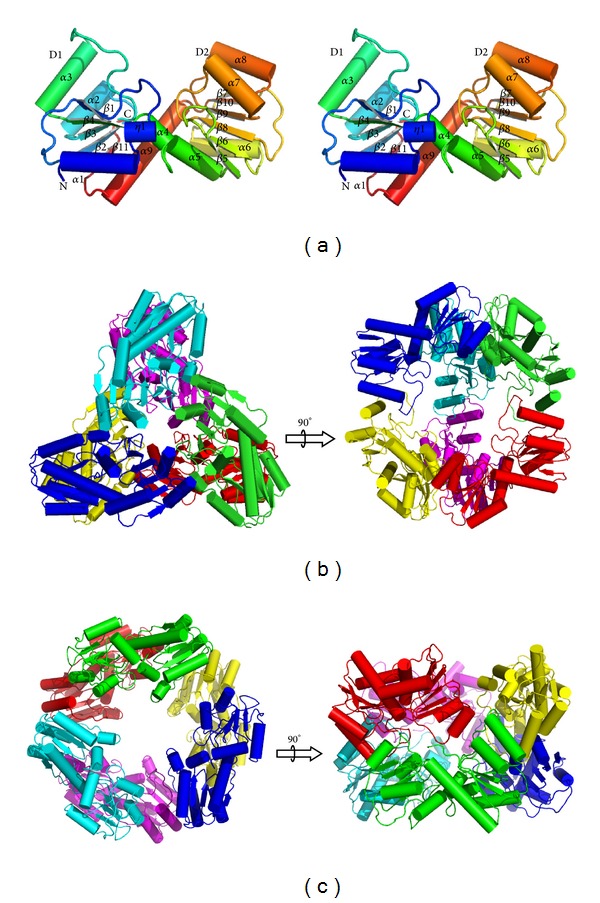
(a) Stereo view of MJ0927 monomer. The protein is rainbow-colored from blue at the N-terminus to red at the C-terminus. (b) The top and side views of the MJ0927 hexamer. The monomers A–F are colored in green, blue, cyan, red, yellow, and magenta, respectively. (c) The top and side views of the TTHA1606 hexamer. Each of the six monomers is in a different color.

Two crystal forms of MJ0927 were obtained, one in the P2_1_ and the other in the C222_1_ space groups. The Se-Met labeled P2_1_ crystal structure was solved at 2.8 Å resolution by using the multiwavelength anomalous diffraction (MAD) method. The refined structure was subsequently used as the template to solve the native C222_1_ structure by a molecular replacement approach. The final model was refined to 2.47 Å resolution with good *R*_work_ and *R*_free_ values. Residues 6–249 of the polypeptide chain were well defined in the electron-density maps, excluding the 5 N-terminal end residues which were invisible. A ribbon representation of a single MJ0927 monomer is shown in Figure 1(a). The P2_1_ crystal form contains six molecules in each asymmetric unit, forming a hexameric spheroid with a 32-symmetry (Figure 1(b)). The six independent molecules in the asymmetric unit superpose well with pairwise root-mean-square deviations (rmsd) ranging from 0.222 to 0.310 Å. The C222_1_ crystal form contains a single copy of a trimer in each asymmetric unit. No significant structural differences of the individual molecules were observed between the P2_1_ and C222_1_ crystal forms.

The overall structure of the MJ0927 monomer resembles a typical SCOP-classified NIF3-like fold [16] and is composed of 11 *β*-strands (*β*1–*β*11), nine *α*-helices (*α*1–*α*9), and one 3_10_-helice. This protein appears to fold into two interlinked *α*/*β* Nif3 domains, each consisting of two *α*-helix layers sandwiching a single *β*-sheet (Figure 1(a)). The first domain (D1) contains a 5-stranded mixed *β*-sheet flanked by two *α*-helices and three *α*-helices on either side. The second domain (D2) is characterized by a central mixed *β*-sheet comprised of six *β*-strands with a pair of *α*-helices on both sides.

**Figure 2:**
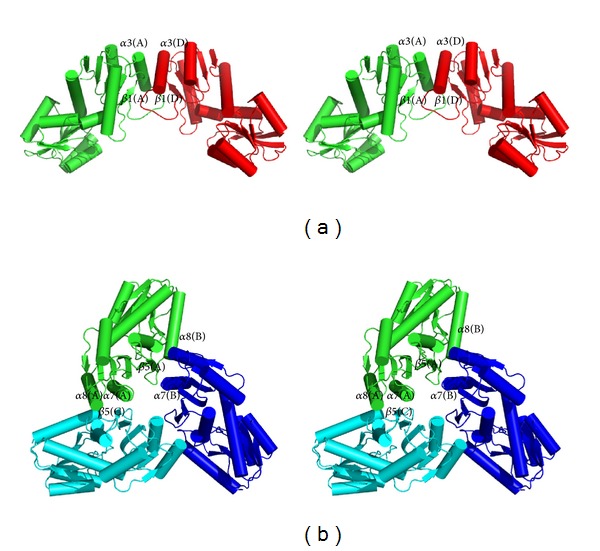
(a) Stereo view of the MJ0927 dimer. (b) A stereo top-view of the MJ0927 trimer along its 3-fold axis.

The MJ0927 hexamer can be described as two stacked trimers, consisting of monomers A-B-C and D-E-F, which are related by a two-fold symmetry (Figure 1(b)). The intratrimer interface comprises two major regions, one consisting of a *β*-strand–helix interaction between *β*-strand *β*5 and helices *α*7 and *α*8 derived from one adjacent subunit, while the other is formed by helices *α*7 and *α*8, and *β*-strand *β*5 derived from another subunit (Figure 2(b)). The surface area of each subunit buried in the intratrimer interface is 1110 Å^2^, representing ~9.1% of the surface area of each subunit. There are five direct hydrogen bonds and six salt bridges and extensive hydrophobic interactions between the monomers across the intratrimer interface (Table 3). Contacts between molecules A and D, B and E, or C and F occur through the interdimer interactions. The surface area of each subunit buried in the interdimer interface is 1207 Å^2^, which constitutes ~10% of each subunit surface area. A total of 21 direct hydrogen bonds and four salt bridges are observed across the interdimer interface of each subunit (Table 2), with significant hydrophobic interactions formed by the helix *α*3, the *β*-strand *β*1, and the loop region L*α*_3-_ *β*_3_ (Figure 2(a)). The hexamer is stabilized by a combination of interdimer and intratrimer interactions. These results are consistent with the previous gel filtration experiment showing that MJ0927 exists as a hexamer in solution [10].

**Figure 3:**
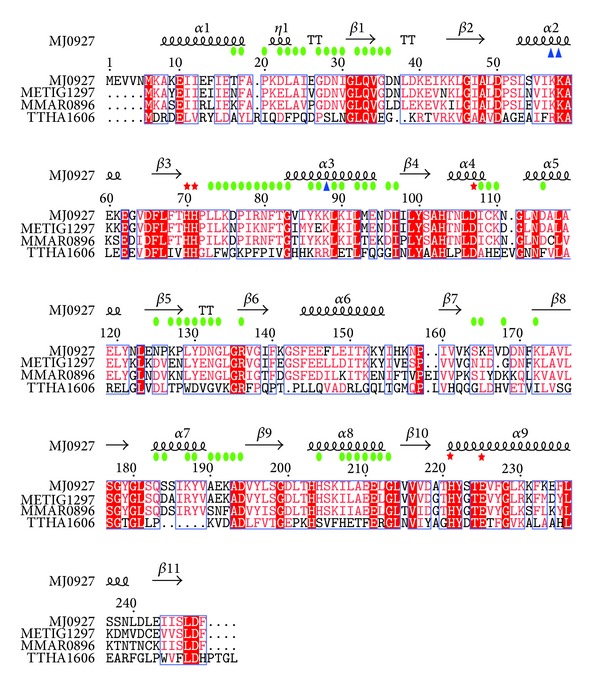
Sequence alignment of MJ0927with* Methanotorris igneus* METIG1297*, Methanococcus maripaludis* MMAR0896, and* Thermus thermophilus *HB8TTHA1606. The following NCBI gi accession numbers reference the sequences used for the alignment: MJ0927, 499172854; METIG1297, 503565355; MMAR0896, 501148655; TTHA1606, 499487006. Sequences are numbered according to MJ0927, and secondary structure elements are displayed above the alignment. Red asterisks indicate metal-binding residues. Blue triangles indicate residues predicted for DNA binding. Green circles indicate residues involved in hexamer formation. Residues that are completely conserved are highlighted in solid red boxes. Those with similarity of >70% are labeled in red. The alignment was generated with the ClustalW2 program [17] (http://www.ebi.ac.uk/Tools/msa/clustalw2/) and used as the input for the ESPript program [18], version 2.2 (http://espript.ibcp.fr/ESPript/cgi-bin/ESPript.cgi).

Since MJ0927 is a thermophilic archaeal Nif3 protein, it is not surprising that the quaternary structure of MJ0927 differs from other bacterial Nif3 proteins with regard to the stabilizing structural elements, particularly the structural arrangement at the intratrimer interfaces. As seen in Figure 2(b), the intratrimer interfaces are tightly packed between the second domains of MJ0927 and the three subunits are arranged in a tail-to-tail manner. To date, trimer contacts in these solved bacterial Nif3 structures are only found in the SA1388 and YqfO [6, 8], which use PII-like domains that appear to cap the openings on either side of the central channel in the toroidal rings. Therefore, the trimer organization of MJ0927 is a unique structural feature that likely contributes to its increased structural stability and which has never been observed in other Nif3 proteins. In addition, a sequence alignment further revealed that the Nif3 members in the* Methanotorris*,* Methanocaldococcus*, and* Methanococcus* species likely exhibit similar hexameric spherical architectures, as residues involved in the hexamer formation are found to be highly conserved (Figure 3). Although the biological function of MJ0927 remains unclear, this sequence analysis suggests that the hexameric spherical structure and its overall shape are preserved during evolution and thus may have functional significance.

Additionally, sequence analysis revealed that the highly conserved metal-binding motifs are located in the cavity between the D1 and D2 domains, which consist of three histidines, one glutamate, and one aspartate (Figure 3). In the three bacterial Nif3 protein structures (YbgI, YqfO, and SA1388) [6–8], two divalent metal ions were found to occupy the metal-binding sites. However, no metal ion was observed in the metal-binding site of MJ0927 even though MJ0927 contains these conserved residues. The absence of endogenous metal ions in MJ0927 is similar to the structures reported for SP1609 and TTHA1606 [9], although the reason for this phenomenon is still unclear.

**Figure 4:**
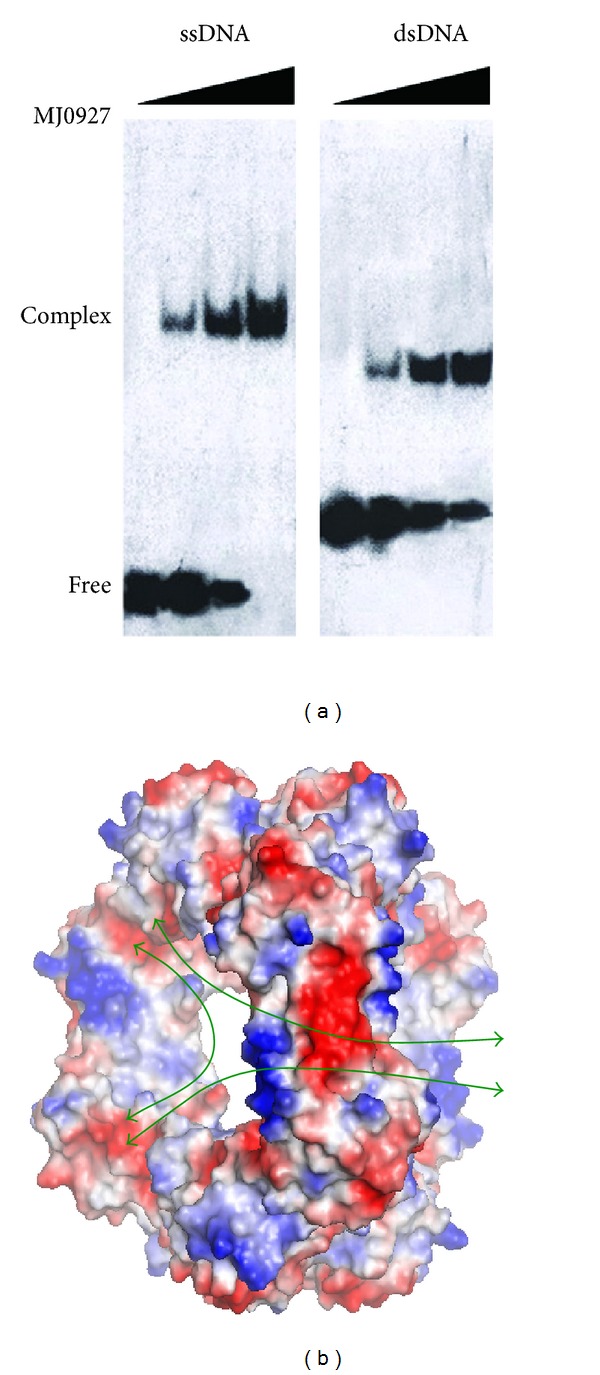
(a) EMSA analysis of MJ0927. 25 nM ssDNA was incubated with various amounts of MJ0927 at 0, 50, 250, and 500 *μ*M in a 10-*μ*L reaction mixture and 25 nM dsDNA was incubated with increasing amounts of MJ0927 at 0, 50, 100, and 200 *μ*M. (b) Surface representations displaying the electrostatic potential of MJ0927. Electrostatic potential was calculated using the program APBS [19] implemented in PYMOL (The PyMOL Molecular Graphics System, Version 0.99rc6 Schrödinger, LLC.). Positive potential is shown in blue, neutral in white and negative potential in red. Green arrows indicate possible paths for DNA entry.

Since TTHA1606 has been shown to bind ssDNA [9], we are also interested to see if MJ0927 were capable of binding DNA. To assess the DNA-binding ability of MJ0927, purified proteins were incubated with ssDNA or dsDNA and the resulting complexes were separated by EMSA. Results of this analysis indicated that MJ0927 indeed is able to bind to both ssDNA and dsDNA (Figure 4(a)). The dsDNA-binding property of MJ0927 was not observed in other Nif3 family proteins reported to date.

No Nif3-DNA complex structure has ever been reported yet. However, structural analyses of MJ0927 could provide some insights as to how the Nif3 protein binds to DNA. From the determined Nif3 structures reported to date, only those of MJ0927 and TTHA1606 were shown to bind ssDNA. Similar to TTHA1606, MJ0927 also possesses positively charged residues clustered on the helices *α*2 and *α*3 near the putative active site. Most of these residues share high sequence similarity between the MJ0927 and TTHA1606 Nif3 proteins, suggesting that the positively charged region may be involved in DNA binding (Figure 2(a)). Furthermore, the quaternary assembly of MJ0927 is significantly different to those previously solved bacterial Nif3 proteins. The bacterial Nif3s except for SA1388 and YqfO consist of three dimers forming toroidal ring quaternary structures, which possess an opened channel in the center of the hexamer (Figure 1(c)), whereas MJ0927 adopts an unusual assembly comprising a trimer of dimers that forms a cage-like architecture (Figure 1(b)). The architecture of MJ0927 leads to three large openings (Figure 4(b)). The diameter of the openings is approximately 33 Å, which is large enough to allow ssDNA or dsDNA entry. The sphere assembly of MJ0927 leads to larger openings than that of TTHA1606. Therefore, MJ0927 binds to both ssDNA and dsDNA, but TTHA1606 only binds to ssDNA.

## 4. Conclusions

MJ0927 is a member of the Nif3 family and is highly conserved among bacteria and humans. Here we describe the crystal structure of MJ0927, revealing an unusual hexameric assembly. Electrophoretic mobility-shift assays indicated that MJ0927 can bind to both ssDNA and dsDNA. The studies presented here clearly indicate that hexameric MJ0927 possesses ssDNA- and ds-DNA-binding properties and helps to further define its function. In a proposed follow-up study, we will try to determine the structure of MJ0927 in complex with DNA with the intent of identifying the DNA-binding residues of this protein to gain insight into its mechanism of action.

## Figures and Tables

**Table 1:** Data collection statistics for the MJ0927 crystal. Values in parentheses are for the highest resolution shell. Data of C222_1_ form has been used in previous publication [10].

	Se-Met Nif3 (P2_1_ form)			Nif3 (C222_1_ form)
Data Collection				
Wavelength (Å)	Peak	Edge	Remote	
	0.9792	0.9793	0.9641	1.0000
Space group		P2_1_		C222_1_
Unit Cell (Å)		95.60, 77.21, 131.85		81.21, 172.94, 147.42
		*β* = 105.36		
Resolution range (Å)	30–2.8 (2.90–2.80)	30–2.7 (2.80–2.68)	30–2.75 (2.85–2.75)	30–2.47 (2.56–2.47)
Total observations	340792 (34238)	189181 (18903)	180913 (18198)	271300 (26582)
Unique reflections	45486 (4505)	51206 (5109)	48495 (4789)	37448 (3692)
Completeness (%)	99.8 (99.8)	99.7 (99.8)	99.7 (99.9)	99.3 (100)
*I*/*σ*〈*I*〉	23.1 (4.4)	16.1 (2.8)	18.2 (2.8)	34.3 (4.6)
*R*_merge_ (%)	5.9 (33.3)	5.1 (33.6)	4.7 (33.3)	5.3 (46.5)
Refinement				
Resolution range (Å)		30–2.68 (2.73–2.68)		30–2.47 (2.53–2.47)
Reflections (*F* > 0*σ*_*F*_)		51170 (2370)		37404 (2689)
*R*_work_ (%) for 90% data		18.8 (26.0)		18.1 (24.1)
*R*_free_ (%) for 10% data		25.7 (33.8)		23.6 (35.8)
RMS deviations				
Bond lengths (Å)		0.008		0.007
Bond angles (°)		1.13		1.08
Average *B*-factors (Å^2^)				
Protein atoms		58.0		49.0
Water molecules		54.2		45.6
Model content				
Protein residues		1464		732
Waters		381		192

**Table 2:** Interacting surfaces in MJ0927 and interactions at the interdimer interface.

Type of contacts	Monomer A	Monomer D
	Residue	Residue
Salt bridges	Asp36	Lys87
	Asp96	Lys90
	Lys87	Asp36
	Lys90	Asp96

Hydrogen bonds	Arg79[O]	Asn29[ND2]
	Phe81[O]	Gly35[N]
	Ile78[O]	Ile78[N]
	Lys75[O]	Arg79[NH1]
	Gly27[O]	Arg79[NH1]
	Gly27[O]	Arg79[NH2]
	Leu32[O]	Asn80[ND2]
	Asn29[OD1]	Asn80[ND2]
	Gln33[O]	Phe81[N]
	Gly35[O]	Thr82[OG1]
	Asp96[OD1]	Tyr86[OH]
	Asp36[OD1]	Lys87[NZ]
	Asn29[ND2]	Arg79[O]
	Gly35[N]	Phe81[O]
	Ile78[N]	Ile78[O]
	Arg79[NH1]	Lys75[O]
	Arg79[NH1]	Gly27[O]
	Arg79[NH2]	Gly27[O]
	Asn80[ND2]	Asn29[OD1]
	Phe81[N]	Gln33[O]
	Tyr86[OH]	Asp96[OD1]

**Table 3:** Interacting surfaces in MJ0927 and interactions at the intratrimer interface.

Type of contacts	Monomer A	Monomer B	Monomer C
	Residue	Residue	Residue
Salt bridges	Asp22	His204	
	Lys192	Glu191	
	Lys127	Asp194	
	Asp194		Lys127
	Glu191		Lys192
	His204		Asp22

Hydrogen bonds	Tyr188[OH]	Glu191[OE1]	
	Lys127[NZ]	Ala193[O]	
	Lys110[N]	Glu211[OE1]	
	Glu211[OE1]		Lys110[N]
	Glu191[OE1]		Tyr188[OH]
